# Effects of Co-application of Cadmium-Immobilizing Bacteria and Organic Fertilizers on *Houttuynia cordata* and Microbial Communities in a Cadmium-Contaminated Field

**DOI:** 10.3389/fmicb.2021.809834

**Published:** 2022-05-06

**Authors:** Xiumei Yu, Min Yan, Yongliang Cui, Zhongyi Liu, Han Liu, Jie Zhou, Jiahao Liu, Lan Zeng, Qiang Chen, Yunfu Gu, Likou Zou, Ke Zhao, Quanju Xiang, Menggen Ma, Shuangcheng Li

**Affiliations:** ^1^College of Resources, Sichuan Agricultural University, Chengdu, China; ^2^Sichuan Provincial Academy of Natural Resource Sciences, Chengdu, China

**Keywords:** bacterial community, cadmium-immobilizing bacteria, cadmium, biofertilizer, *Houttuynia cordata*

## Abstract

Cadmium pollution is a serious threat to the soil environment. The application of bio-based fertilizers in combination with beneficial microbial agents is a sustainable approach to solving Cd pollution in farm soil. The present study investigated the effects of co-application of a Cd-immobilizing bacterial agent and two fermented organic fertilizers (fermentative edible fungi residue; fermentative cow dung) on *Houttuynia cordata* and its microbial communities in a Cd-polluted field. It showed that both the application of the Cd-immobilizing bacterial agent alone and the combined application of bio-based soil amendments and the bacterial agent effectively reduced >20% of the uptake of Cd by the plant. Soil nitrogen level was significantly raised after the combined fertilization. The multivariate diversity analysis and co-occurrence network algorithm showed that a significant shift of microbial communities took place, in which the microbial populations tended to be homogeneous with reduced microbial richness and increased diversity after the co-application. The treatment of fermentative cow dung with the addition of the bacterial agent showed a significant increase in the microbial community dissimilarity (*R* = 0.996, *p* = 0.001) compared to that treated with cow dung alone. The co-application of the bacterial agent with both organic fertilizers significantly increased the abundance of *Actinobacteria* and *Bacteroidetes*. The FAPROTAX soil functional analysis revealed that the introduction of the microbial agent could potentially suppress human pathogenic microorganisms in the field fertilized with edible fungi residue. It also showed that the microbial agent can reduce the nitrite oxidation function in the soil when applied alone or with the organic fertilizers. Our study thus highlights the beneficial effects of the Cd-immobilizing bacterial inoculant on *H. cordata* and provides a better understanding of the microbial changes induced by the combined fertilization using the microbial agent and organic soil amendments in a Cd-contaminated field.

## Introduction

Metallic elements are a naturally occurring and indispensable component of different soil environments (Kabata-Pendias, 1993). Most of these metals are innocuous and pose no threat to vegetation and soil organisms. However, heavy metals such as arsenic (As), cadmium (Cd), chromium (Cr), mercury (Hg), and lead (Pb) can cause serious hazards to soil ecosystem and health, which could further affect human health and well-being by means of bioaccumulation affects through food chain (Liu X. et al., 2013; Osman et al., 2021). One of the worst health-threatening heavy metal that has been polluting arable land and causing global concerns is cadmium (Cd) (Satarug et al., 2003). Cd in soil often occurs in high concentrations, inevitably posing a risk to the health of humans and animals that reside in the immediate vicinity (Schoeters et al., 2006). Cd is exceedingly bioavailable to both plant and animal cells and the toxicity of Cd can be summarized as interference with essential metal uptake in cells (Martelli et al., 2006). Since Cd is not a naturally-existing metal in organisms, the major sources of Cd pollution are anthropogenic activities (Yuan et al., 2019). These Cd pollution causes include burning of fossil fuels, mining and smelting of ore minerals, and inadequate disposal of electronic wastes (Sun et al., 2010; Li et al., 2011; Gujre et al., 2021). It is estimated that more than 5.6–38 kt of Cd is released into the environment per annum and approximately 20% of China’s agricultural soil accounting for 1.3 × 10^5^ ha of the total polluted lands is contaminated with this heavy metal (Rafiq et al., 2014; Liu et al., 2020). Therefore, it is urgent that remediation efforts come into effect for the alleviation of this imminent environmental threat.

Although Cd pollution in soils is known to have a negative impact on the growth and behavior of indigenous plants as well as on the composition and ecological succession of their associated microbial communities (Tiwari and Lata, 2018), beneficial microbial consortia can lead to an improved rhizosphere microenvironment which could help mitigate the negative effects upon plants caused by heavy metal stress (Wenzel, 2009; Kang et al., 2018). There are various ways by which Cd pollution in soils can be alleviated. One classic example is the plant’s secretion of root exudates, which mainly contain primary metabolites and can reduce Cd toxicity in the rhizosphere by absorption, chelation, and complexation (Dong et al., 2007; Canarini et al., 2019). Studies showed that these root-secreted phytochemicals were able to ameliorate Cd tolerance in plants under metal-stressed conditions (Bali et al., 2020). However, these chemicals alone can only play a small part in tackling Cd pollution in soil when compared to the approaches using beneficial microorganisms, which are essentially probiotics for plants (Pramanik et al., 2018; Konkolewska et al., 2020). A previous study showed that the application of a plant growth-promoting rhizobia inoculant alleviated stresses to native plants caused by the presence of high levels of toxic heavy metals in multimetallic tailing soil (Kang et al., 2018). Another recent research demonstrated that the application of beneficial *Pseudomonas* strains in the soil mitigated the lethal effects of chromium and significantly improved the growth of *Abelmoschus esculentus* (Mushtaq et al., 2021). In addition, highly soluble metals such as Cd and Cu can be immobilized by plant growth-promoting strains, leading to less metals to be transferred to the above-ground parts of plants (Ke et al., 2021). Recent studies showed that the most important mechanisms of Cd immobilization in soil by microorganisms are microbially induced carbonate precipitation (MICP) and microbially induced phosphate precipitation (MIPP), by which free Cd^2+^ ions in the soil were immobilized after contacting the microbially produced carbonate and various phosphate anions (Fang et al., 2021; Zeng et al., 2021).

Microbial communities are very sensitive to changes in the microenvironments of both rhizosphere and bulk soils in the heavy metal-polluted environment (Yu et al., 2019; Kang et al., 2020; Barra Caracciolo and Terenzi, 2021). Studies indicated that highly toxic metals/metalloids such as As, Cr, Hg, and Pb in the soil can lead to intense changes in the community composition and microbial diversity and cause obstructions to adaptation processes resulting in the decline of microbial populations in metal-polluted ecosystems (Pan et al., 2021; Shuaib et al., 2021). The application of bio-based fertilizers (BBF) in itself is in conformity with circular economy and can mitigate heavy metal pollution in agricultural soil (Huang et al., 2016; Chojnacka et al., 2020; Dhaliwal et al., 2020). In one case, it was reported that the application of metal-immobilizing organic fertilizer led to improved soil quality and more than 50% reduction of extractable Cd, Cu, and Pb (Hu et al., 2021). Other researchers found that bio-based soil amendments decreased soil exchangeable Cd and Cd accumulation in grain, increased soil microbial diversity and species richness, and contributed to faster nutrient turnover (Gong et al., 2021).

However, there is a lack of information on how the microbial communities change with combined application of bio-based fertilizers and beneficial microbial inoculant in a heavy metal-contaminated field with the cultivation of crops. It is also unclear about the *in situ* effects of the combined fertilizers on the growth of plants. Therefore, the present study aims to elucidate the variations in soil microbial communities caused by the field utilization of two bio-based fertilizers and a Cd-immobilizing microbial inoculant, and also to examine the positive resulting effects on *Houttuynia cordata*, which is an important edible and medicinal plant.

## Materials and Methods

### Soil Sampling and Measurements

A cadmium-contaminated field on the Chengdu Plain, Southwest China, was selected for the cultivation of *H. cordata*. The soil type of this field was paddy soil. Soil samples were collected for measurements of cadmium and physicochemical properties and isolation of Cd-immobilizing bacteria.

Soil samples were mixed into slurries with a 2:5 (w/v) soil-water ratio and the pH was then determined using a standard glass/calomel electrode (Shanghai Yoke, China). Air-dried soil samples were digested using a mixture of H_2_SO_4_, H_2_SO_4_-HClO_4_, and HF-HClO_4_ solutions before the concentrations of soil total nitrogen (TN), phosphorus (TP) and potassium (TK) were measured using the Kjeldahl method (Kjeltec 8400, FOSS, Sweden), the Mo-Sb colorimetric method at OD_660nm_ (WFJ2100, UNICO, China), and flame spectrophotometry (FP6410, Shanghai Precision & Scientific, China), respectively (Yu et al., 2017; Kang et al., 2020). Soil organic matter (TO) was determined using the Walkley–Black chromic acid wet oxidation method (Storer, 1984). Soil available phosphorus (AP) and potassium (AK) were simultaneously extracted using the ASI soil extracting solution (Bogunovic et al., 2017), and their concentrations were measured with a UV-3300 spectrophotometer (MAPADA, Shanghai, China) and a FP6410 flame spectrophotometer (PRECISION & SCIENTIFIC, Shanghai China), respectively. Available nitrogen (AN) was determined using the Alkali N-proliferation method (Dahnke and Johnson, 1990). Soil samples, fermentative edible fungi residue, and fermentative cow dung were digested using a mixture of HNO_3_-HCl-HClO_4_ in a 1:2:2 (v:v:v) ratio for the determination of total Cd. The bioavailable fraction of Cd was extracted from the soil samples using pentetic acid (DTPA) (Massas et al., 2013). The concentration of Cd was measured using inductively coupled plasma atomic emission spectroscopy (ICP-AES) (IRIS Intrepid II; Thermo Electron Corporation, San Diego, CA, United States) as described before.

### Isolation of Cadmium-Immobilizing Bacteria

Bacteria were isolated from the field soil samples by using beef extract-peptone agar medium (Yu et al., 2014). The tolerance of bacteria to Cd was represented by the minimum lethal concentration (MLC) of Cd for these isolates. Then, some bacteria with tolerance against Cd were selected to determine Cd^2+^ immobilization using the previously published method (Li et al., 2019). The bacterial strains with an outstanding immobilizing ability were identified by 16S rRNA gene sequencing and genetic affinity analysis (Yu et al., 2014).

### Experimental Design

This research was performed at the *H. cordata* main production field of Chengdu Plain from spring to summer. When *H. cordata* plants emerged as ∼10 cm height seedlings, the field with uniform plant growth was selected to perform six different fertilization treatments. Each treatment had six replications, and each replication of the field had an area of approximately 3 × 5 m^2^. A one-meter-width isolation zone was set between each treatment plot. These six treatment groups were composed of non-fertilization (CK), Cd-immobilizing bacteria agent (MB), fermentative edible fungi residue mixed with Cd-immobilizing bacteria agent (FEMB), fermentative edible fungi residue (FE), fermentative cow dung mixed with Cd-immobilizing bacteria agent (FCMB), and fermentative cow dung (FC). For MB treatment, the mixture of Cd-immobilizing bacteria culture medium (approximately 10^9^ cells/ml) was applied at 120 l/hm^2^ with 400-fold dilution. For the treatments of FEMB and FCMB, the Cd-immobilizing bacteria culture medium was mixed into the fermentative edible fungi and cow dung, with the same application amount of bacteria cells as in MB treatment. The organic fertilizer treatments (FE, FC, FEMB, and FCMB) were applied at 45 t/hm^2^ to the field. Then, the experimental field was conformably managed by the conventional field methods, such as watering during drought.

After 6 months of cultivation, *H. cordata* plant samples and rhizosphere soil samples were collected from each treatment area in the field. Plant and soil samples were randomly collected from five points at each treatment area and separately packed into a sterile plastic bags. Afterward, the soil samples were mixed well, transferred into a sterile EP tube, and immediately frozen using liquid nitrogen. The frozen soil samples were then taken to lab in liquid nitrogen and stored at −80°C for extraction of total soil DNA. The remaining soil samples still kept in bags were taken to lab for air-drying, grounding, screening, and measurements of soil physical and chemical properties and heavy metal contents. The plant samples were taken to lab for the measurement of Cd content. As a result, a total of 36 plant and soil samples were collected for the six treatments. Soil samples were used for measurements of cadmium and physicochemical properties by using the above-mentioned method and extraction of soil total DNA for high-throughput sequencing.

### Analyses of Plant Cadmium and Nutrient Contents

To remove Cd absorbed on root surface, the roots of the harvested *H. cordata* were washed using tap water and, after being immersed in 10 mM EDTA for 30 min, rinsed thoroughly with deionized water (Luo et al., 2011). The roots and aboveground parts of *H. cordata* plants were separately kept in two envelopes for the deactivation of enzymes at 105°C for 30 min and at 80°C until constant weight in an oven. The oven-dried roots and aboveground parts were triturated and digested with HNO_3_/HClO_4_ solution (4:1, v/v). The Cd contents in roots and aboveground parts were measured with inductively coupled plasma atomic emission spectroscopy (ICP-AES, IRIS Intrepid II; Thermo Electron) (Kang et al., 2018). The contents of nitrogen, phosphorus and potassium in *H. cordata* plant tissues were determined using the commonly used methods (Thomas et al., 1967).

### Extraction of Soil Total DNA

A precise amount of 0.5 g of the frozen soil was taken from EP tubes for the extraction of soil total DNA using FastDNA*™* SPIN Kit (MP Biomedicals LLC, Santa Ana, CA, United States). A soil sample was put into a sterile EP tube and mixed with phosphate buffer (978 μl) and MTbuffer (122 μl). The tube was shaken for 30 min, and then centrifuged at 14,000 r min^–1^ for 15 min. The supernatant was transferred into another sterile EP tube which was filled with 250 μl 10 × PPS. The mixture was centrifuged at 14,000 r min^–1^ for 5 min to remove the precipitation and the resulting supernatant was transferred into another sterile EP tube. After adding the adsorption suspension (1 ml), the supernatant mixture was precipitated onto the SPIN filter membrane followed by centrifugation at 12,000 r min^–1^ for 1 min to collect total DNA from the membrane. After this, the SPIN filter membrane was washed by adding ethanol liquid and centrifugation at 12,000 r min^–1^ for 1 min. Total DNA on the membrane was dissolved into 50 μl ddH_2_O by centrifugation at 14,000 r min^–1^ for 1 min after being properly air-dried. The quality of the extracted soil total DNA was tested using polyacrylamide gel electrophoresis (PAGE) and a NanoDrop ND1000 spectrophotometer (NanoDrop Technologies, Wilmington, DE, United States) at wavelengths of 260/280 and 260/230 nm. The eligible total DNA was stored at −80 °C.

### Amplicon Library Construction and High-Throughput Sequencing

High-quality soil total DNA was used for the high-throughput sequencing of the V3–V4 hyper variable region of prokaryotic 16S rRNA using the universal primers 515F and 806R (Wu X. et al., 2016). PCR reaction was performed in a reaction mixture (30 μl) containing 15 μl Phusion^®^ High-Fidelity PCR Master Mix (New England Biolabs), 0.2 μM primers and 10 ng template DNA. Thermal cycling conditions consisted of initial denaturation at 98°C for 1 min, 30 cycles of denaturation at 98°C for 10 s, annealing at 50°C for 30 s, elongation at 72°C for 30 s, and a final extension step of 72°C for 5 min. The amplicons with a bright main strip between 400 and 450 bp were selected using electrophoresis on 2% agarose gel for sequencing library preparation, which was achieved using NEB Next^®^ Ultra*™* DNA Library Prep Kit for Illumina (NEB, Ipswich, MA, United States) following manufacturer’s instructions. The quality of the amplicon library was assessed using a Qubit@ 2.0 Fluorometer (Thermo Scientific) and Agilent Bioanalyzer 2100 system. The amplicon library was sequenced on the Illumina HiSeq platform (Illumia, San Diego, CA, United States) (Caporaso et al., 2012).

### Bioinformatics and Statistical Analyses

The raw sequences were analyzed using QIIME2 2019.10 workflow. The paired-end reads were first assembled using USEARCH algorithm and those with the Phred quality scores less than 4 or with ambiguous bases were removed before further analysis. The clean sequences were subsequently denoised using deblur algorithm, in which all sequences were trimmed to a length of 350 bp. Given the biases introduced by primers, the first 35 bp of all sequences was trimmed. The amplicon variant sequences (ASVs) produced after denoising were aligned in Silva 132 database using classify-sklearn algorithm to infer their taxonomic classifications. To ensure the accuracy of final results, singletons and sequences classified as mitochondria or chloroplast were filtered before all samples were rarefied to 8,388 sequences. A table containing ASVs were thus created for subsequent diversity analyses. Functional Annotation of Prokaryotic Taxa (FAPROTAX) (Louca et al., 2016) was used to predict the biogeochemical functions of communities, and the functions with significant (*p* < 0.05) differences between treatments were demonstrated on the graphs.

The α diversity indices calculated by QIIME2 workflow were compared using Wilcoxon rank sum test (*p* < 0.05). Bacterial composition in each treatment was visualized using ‘ggplot2’ in R Studio. To reveal the differences in bacterial composition among six treatments, the Bray–Curtis distance matrices of microbial communities in each treatment were created and subsequently compared through the analysis of similarity (ANOSIM) using ‘vegan’ package. The ‘DESeq2’ package (Love et al., 2014) was used to identify differential bacteria among treatments by calculating the enrichment and depletion of ASVs between two treatments (log2 fold changes = 1, FDR < 0.05) (Li F. et al., 2017), and the results generated by this analysis were visualized as volcano diagrams. The LDA effect size (LEfSe) analysis was applied to estimate the differential bacteria at taxonomic levels (LDA score > 4, *p* < 0.05) and the differences in biogeochemical functions (LDA score > 1.5, *p* < 0.05) using ‘microeco’ package (Liu et al., 2021) hosted on the Microbiome Database^1^. Significant biogeochemical functions were further visualized using ‘pheatmap’ package. To reveal the relationships between the shifts in bacterial communities and the differences of micro-habitats across six treatments, we performed the redundancy analysis (RDA) based on Bray–Curtis distance using ‘vegan’ package. To reveal the bacterial relationships in each treatment, six co-occurrence networks at genus level were created using ‘igraph’ package (Csardi and Nepusz, 2006) (Spearman ρ = 0.6, *p* < 0.05) and the results were further visualized using Gephi v0.9.2^2^ (Yu et al., 2019; Kang et al., 2020).

## Results

### Soil Properties and Cd-Immobilizing Bacterial Strains

Soil in the *H. cordata* field contained 0.58 ± 0.04 mg kg^–1^ of total Cd and 0.15 ± 0.01 mg kg^–1^ of bioavailable Cd, which exceeded the standard value stipulated in the Soil Environmental Quality Standard of China. The sample contained a high level of organic matter (10.46% ± 0.13%) with low pH (5.61 ± 0.18). The total contents of nitrogen (N), phosphorus (P), and potassium (K) were 1.87 ± 0.23 g kg^–1^, 0.93 ± 0.13 g kg^–1^, and 10.62 ± 0.53 g kg^–1^, whereas the bioavailable fractions were 185.30 ± 7.93 mg kg^–1^, 77.63 ± 2.26 mg kg^–1^, 139.50 ± 3.33 mg kg^–1^, respectively.

Four bacterial strains with an outstanding immobilizing ability were isolated from the cadmium-contaminated farm field soil. According to 16S rRNA genetic affinity analysis, the Cd-immobilizing bacterial strains GH8-1 and ZJ1-16B were identified as *Bacillus iocasae*, WDGJ-1 as *Bacillus subtilis*, and NQ5-6 as *Bacillus wiedmannii*. Since these four Cd-immobilizing bacterial strains had no antagonism against each other, they were mixed in a ratio of 1:1:1:1 and applied into the treatments of MB, FEMB, and FCMB in the *H. cordata* field experiment.

### Cd and Nutrient Contents in *H. cordata*

After a total of 6 months of cultivation, the *H. cordata* showed vigorous growth without significant difference in fertilization treatment groups, but the fertilized plants looked better than the control plants. Cd absorption effect by this plant was observed when grown in Cd-contaminated soil. The enrichment of Cd in roots and aboveground parts of *H. cordata* was significantly different (*p* < 0.05) under different fertilization treatments (Table 1). The Cd content in the roots of *H. cordata* was about four to six times higher than that in the aboveground parts. Compared to the control (CK), both the fermentative edible fungi residue (FE) and fermentative cow (FC) caused approximately 30% and 32% increases of Cd content in roots and 11% and 27% increases of Cd in aboveground parts, respectively. The introduction of the Cd-immobilizing bacteria reduced Cd content in roots by 25% and in aboveground parts by 17%. When the Cd-immobilizing bacterial inoculant was mixed with fermentative edible fungi residue (FEMB) and fermentative cow dung (FCMB), a decrease of Cd content was induced in roots by 22% and in aboveground parts by 24%.

**TABLE 1:** Contents of nitrogen, phosphorus, potassium, and cadmium in roots and shoots of *Houttuynia cordata*.

Measured parameter	Plant tissue	CK	MB	FE	FEMB	FC	FCMB
Cd (mg kg^–1^)	Roots	1.86 ± 0.05b***^a^***	1.39 ± 0.23c***^b^***	2.46 ± 0.26a*^a^*	1.92 ± 0.14ab*^b^*	2.41 ± 0.23a**^b^**	1.84 ± 0.16ab**^a^**
	Aboveground parts	0.30 ± 0.03c***^a^***	0.36 ± 0.03bc***^a^***	0.40 ± 0.01ab*^a^*	0.33 ± 0.02bc*^b^*	0.46 ± 0.06a**^a^**	0.36 ± 0.07bc**^b^**
TN (g kg^–1^)	Roots	8.30 ± 0.92ab***^a^***	8.30 ± 1.44ab***^a^***	7.20 ± 2.79bc*^a^*	4.44 ± 2.30c*^a^*	9.85 ± 1.05ab**^a^**	10.51 ± 0.49a**^a^**
	Aboveground parts	11.95 ± 1.46a***^b^***	14.97 ± 1.71a***^a^***	13.63 ± 1.83a*^a^*	11.64 ± 2.62a*^a^*	13.82 ± 1.89a**^a^**	12.41 ± 2.63a**^a^**
TP (g kg^–1^)	Roots	24.21 ± 3.29a***^a^***	11.45 ± 2.68b***^b^***	11.70 ± 3.52b*^a^*	11.03 ± 2.28b*^a^*	22.77 ± 4.44a**^a^**	11.33 ± 1.78b**^b^**
	Aboveground parts	23.48 ± 2.10a***^a^***	21.73 ± 1.37a***^a^***	21.27 ± 3.88a*^a^*	22.64 ± 2.50a*^a^*	13.78 ± 3.96b**^a^**	9.83 ± 2.17b**^a^**
TK (g kg^–1^)	Roots	34.17 ± 1.50a***^a^***	33.90 ± 1.69a***^a^***	33.95 ± 3.65a*^a^*	33.35 ± 1.50a*^a^*	33.28 ± 1.19a**^a^**	30.28 ± 1.95a**^b^**
	Aboveground parts	28.93 ± 0.70b***^a^***	29.47 ± 0.62b***^a^***	31.38 ± 0.97a*^a^*	28.78 ± 1.40b*^b^*	31.60 ± 1.07a**^a^**	31.60 ± 0.81a**^a^**

*Different lowercase letters indicate significant differences (p < 0.05). Italic bold superscript letters indicate differences between CK and MB; italic superscript letters indicate differences between FE and FEMB; bold superscript letters indicate differences between FC and FCMB. Conventional letters indicate differences of the same measured parameter among all treatments.*

As the three major nutrient elements of plants, the concentrations of nitrogen, phosphorus, and potassium in roots and aboveground parts of *H. cordata* grown in differently treated soils were significantly different (Table 1). For nitrogen, the concentration in roots was generally lower than that in aboveground parts. Nitrogen content was highest in the roots of *H. cordata* from the soil treated with FCMB, whereas it was not significantly different (*p* < 0.05) in the aboveground parts of the plant in all treatment groups. The phosphorus content in the roots of *H. cordata* grown in CK and FC were double the amount in other field soil samples. The phosphorus concentration in the aboveground parts of *H. cordata* in the soil treated with FC and FCMB was significantly lower (*p* < 0.05) than that in the other four treatment groups. The potassium concentrations showed no significant difference (*p* < 0.05) among all treatment groups. However, potassium in the aboveground parts of *H. cordata* in the soil treated with FE, FC, and FCMB was significantly higher (*p* < 0.05) than that in other three treatment groups.

### Cd Content and Physicochemical Properties in the Substrates

There was no significant change in the soil total Cd content in the field grown with *H. cordata* compared to the background total Cd content after 6 months with different fertilizer regimens (Table 2). However, the content of bioavailable Cd in different fertilizer treatment groups was significantly different. Especially, the concentration of bioavailable Cd in FEMB and FCMB-treated soils significantly decreased by 18% compared to CK. We also measured the Cd concentration in the organic fertilizers, which showed that the fermentative fungi residue (FE) contained 0.78 mg kg^–1^ total Cd, while the cow dung (FC) contained 0.56 mg kg^–1^ total Cd. There was 0.15 mg kg^–1^ Cd in FE and 0.14 mg kg^–1^ Cd in FC. Soil pH in CK was not significantly altered after Cd-immobilizing bacteria was added alone, indicating that the addition of Cd-immobilizer on its own could not change soil pH. However, soil pH was significantly increased in the treatments of FE and FC, while it was significantly decreased in FCMB. Although organic fertilizers had been applied in some treatment groups, soil organic matter (TO) in all the treatment groups did not significantly differ from that in CK after 6 months (Table 2). The available nitrogen (AN) in MB was significantly increased (*p* < 0.05) by 16% compared to CK, whereas that in other treatment groups was significantly reduced (*p* < 0.05) (Table 2). Available phosphorus (AP) concentration in other fertilizer-treated groups was significantly higher (*p* < 0.05) than that in CK. The concentration of available potassium (AK) in non-fertilized soil was the highest, whereas that in MB was the lowest among all the treatments. The concentrations of total potassium (TK) and total nitrogen (TN) in all treatment groups was not significantly different (*p* < 0.05). Total phosphorus (TP) was the highest in FEMB, followed by FEMB and the other three treatment groups.

**TABLE 2:** Soil cadmium content and physicochemical properties.

Soil property	CK	MB	FE	FEMB	FC	FCMB
ACd (mg kg^–1^)	0.17 ± 0.01ab***^a^***	0.15 ± 0.01bc***^a^***	0.18 ± 0.01a*^a^*	0.14 ± 0.01c*^b^*	0.16 ± 0.01bc**^a^**	0.14 ± 0.02c**^a^**
TCd (mg kg^–1^)	0.59 ± 0.03a***^a^***	0.57 ± 0.04a***^a^***	0.58 ± 0.03a*^a^*	0.61 ± 0.03a*^a^*	0.59 ± 0.05a**^a^**	0.58 ± 0.02a**^a^**
pH	5.85 ± 0.08bc***^a^***	5.88 ± 0.09bc***^a^***	6.10 ± 0.20a*^a^*	5.94 ± 0.09abc*^a^*	6.05 ± 0.08ab**^a^**	5.78 ± 0.03c**^b^**
TO (%)	9.84 ± 0.41ab***^a^***	10.48 ± 0.68a***^a^***	10.39 ± 0.22a*^a^*	9.73 ± 0.44ab*^b^*	10.36 ± 0.42ab**^a^**	9.55 ± 0.21b**^b^**
AN (mg kg^–1^)	216.83 ± 6.29b***^b^***	252.47 ± 8.50a***^a^***	195.73 ± 9.32c*^a^*	204.13 ± 4.88bc*^a^*	195.73 ± 4.76c**^b^**	207.10 ± 7.47bc**^a^**
AP (mg kg^–1^)	82.04 ± 3.78d***^a^***	86.48 ± 6.10cd***^a^***	95.19 ± 1.66abc*^a^*	87.65 ± 4.08bcd*^b^*	96.11 ± 6.04ab**^a^**	102.04 ± 4.32a**^a^**
AK (mg kg^–1^)	210.83 ± 8.81a***^a^***	101.17 ± 6.87d***^b^***	132.50 ± 5.44c*^b^*	141.33 ± 4.82c*^a^*	105.33 ± 5.22d**^b^**	184.33 ± 7.91b**^a^**
TN (g kg^–1^)	2.21 ± 0.27a***^a^***	2.52 ± 0.52a***^a^***	2.65 ± 0.14a*^a^*	2.74 ± 0.43a*^a^*	2.30 ± 0.20a**^b^**	2.94 ± 0.59a**^a^**
TP (g kg^–1^)	1.01 ± 0.07b***^a^***	1.04 ± 0.08b***^a^***	1.09 ± 0.14ab*^a^*	1.26 ± 0.09a*^a^*	1.01 ± 0.07b**^a^**	1.06 ± 0.16ab**^a^**
TK (g kg^–1^)	11.09 ± 0.63a***^a^***	11.29 ± 0.78a***^a^***	11.30 ± 0.78a*^a^*	11.49 ± 0.58a*^a^*	10.94 ± 0.54a**^a^**	11.47 ± 0.91a**^a^**

*Different lowercase letters indicate significant differences (p < 0.05). Italic bold superscript letters indicate differences between CK and MB; italic superscript letters indicate differences between FE and FEMB; bold superscript letters indicate differences between FC and FCMB. Conventional letters indicate differences of the same test index among all treatments.*

### Abundance of Bacterial Communities Under Different Fertilization Regimens

A total of 3,304,373 reads of the partial 16S rRNA gene sequences were obtained from the high throughput sequencing for the 42 soil samples. After quality filtering and removal of unique tags, the raw sequences yielded 352,296 of qualified sequences, which were finally clustered into 8388 amplicon sequence variants (ASVs) for the following analyses. The α-diversity indices showed significant changes (*p* < 0.05) in bacterial community richness but no obvious shifts in community diversity among the six treatment groups (Table 3).

**TABLE 3:** Alpha-diversity of bacteria communities in the soil grown with *Houttuynia cordata* under different fertilization regimens.

Treatment	CK	MB	FEMB	FE	FCMB	FC
Observed ASVs	2011 ± 65ab***^a^***	2001 ± 149ab***^a^***	1898 ± 180b*^a^*	1885 ± 208b*^a^*	2022 ± 150ab**^a^**	2193 ± 126a**^a^**
Good’s coverage	0.92 ± 0.01ab***^a^***	0.92 ± 0.01ab***^a^***	0.94 ± 0.02a*^a^*	0.93 ± 0.02a*^a^*	0.93 ± 0.02ab**^b^**	0.90 ± 0.01b**^a^**
Chao1	2477 ± 162ab***^a^***	2474 ± 259ab***^a^***	2199 ± 329b*^a^*	2200 ± 361b*^a^*	2354 ± 312ab**^b^**	2824 ± 270a**^a^**
ACE	2657 ± 171ab***^a^***	2668 ± 283ab***^a^***	2318 ± 363b*^a^*	2355 ± 410ba*^a^*	2509 ± 360ab**^b^**	3019 ± 302a**^a^**
Shannon	9.82 ± 0.11a***^a^***	9.75 ± 0.21a***^a^***	9.80 ± 0.24a*^a^*	9.72 ± 0.34a*^a^*	10.03 ± 0.10a**^a^**	10.00 ± 0.13a**^a^**
Simpson	0.997 ± 0.001a***^a^***	0.996 ± 0.001a***^a^***	0.996 ± 0.002a*^a^*	0.996 ± 0.002a*^a^*	0.998 ± 0.000a**^a^**	0.997 ± 0.000a**^a^**

*Different lowercase letters indicate significant differences (p < 0.05); italic bold superscript letters indicate differences between CK and MB; italic superscript letters indicate differences between FE and FEMB; bold superscript letters indicate differences between FC and FCMB. Conventional letters indicate differences of the same index among all treatments.*

**FIGURE 1:**
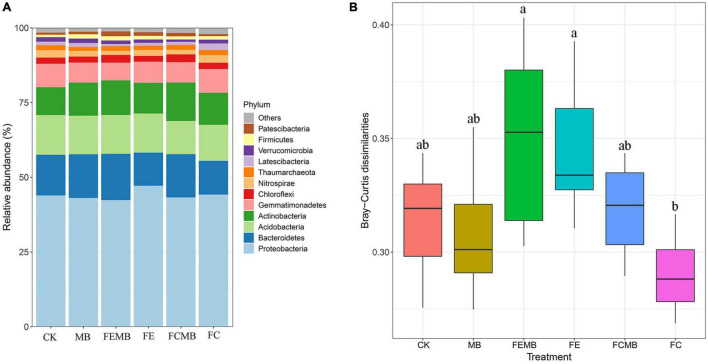
Bacterial community composition in the field grown with *Houttuynia cordata* under different fertilization regimens. **(A)** Relative abundance heatmap of the top 12 phyla. **(B)** Dissimilarity distance of the bacterial community structures; different lowercase letters indicate significant differences (*p* < 0.05).

The abundance analysis revealed that *Proteobacteria* accounted for 42–47% of the entire communities throughout the six groups of fertilizer-treated soils (Figure 1A), indicating that *Proteobacteria* was the dominant phylum in the soil grown with *H. cordata*. However, the shift of bacterial communities in different treatment groups was very obvious. The top 12 phyla in CK were *Proteobacteria* (43.92%), *Bacteroidetes* (13.61%), *Acidobacteria* (13.27%), *Actinobacteria* (9.3%), *Gemmatimonadetes* (7.87%), *Chloroflexi* (2.06%), *Nitrospirae* (2.49%), *Thaumarchaeota* (1.66%), *Latescibacteria* (1.18%), *Verrucomicrobia* (1.49%), *Firmicutes* (0.82%), and *Patescibacteria* (0.72%). Compared with the non-fertilization group, Cd-immobilizing bacterial inoculant increased the relative abundance of *Bacteroidetes*, *Actinobacteria*, *Latescibacteria*, *Firmicutes*, and *Patescibacteria* by 7.36, 18.95, 12.25, 78.99, and 18.01%, respectively. When the inoculant was mixed with the fermentative edible fungi residue, the relative abundance of *Bacteroidetes*, *Actinobacteria*, *Chloroflexi*, *Thaumarchaeota*, *Verrucomicrobia, Firmicutes*, and *Patescibacteria* was increased by 39.78, 12.39, 32.44, 8.32, 5.36, 17.61, and 32.35%, respectively, in the soil grown with *H. cordata*. When Cd-immobilizing bacterial agent was applied in combination with the fermentative cow dung, only *Bacteroidetes*, *Actinobacteria*, *Chloroflexi*, and *Patescibacteria* were increased by 27.76, 20.25, 23.49, and 63.22%, respectively. Under the condition that Cd-immobilizing inoculant was applied alone or combined with fermentative edible fungi residue and cow dung, *Bacteroidetes* and *Patescibacteria* were increased, but *Proteobacteria*, *Acidobacteria*, *Gemmatimonadetes*, and *Nitrospirae* were reduced in different degrees.

The pairwise ANOSIM analysis (Supplementary Table 1) showed that the bacterial communities of CK were significantly separated (*p* < 0.05) from the other five treatment groups. The bacterial communities in the soil where the Cd-immobilizing bacterial inoculant was applied alone were completely different (*p* < 0.05) from those in the other four treatment groups. The bacterial community dissimilarity between FCMB and FC was significant (*R* = 0.996, *p* = 0.001), whereas no obvious difference was found between FEMB and FE (*R* = 0.154, *p* = 0.094). The dissimilarity distance plot (Figure 1B) also showed that the difference in bacterial community structure between FCMB and FC was the highest while that between FEMB and FE was unapparent.

### Differences Between Bacterial Communities Under Different Fertilization Regimens

**FIGURE 2:**
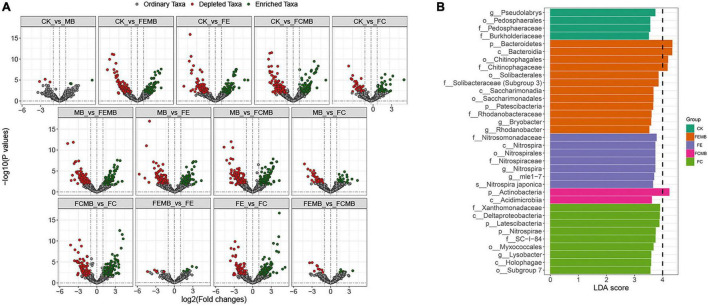
Significance and differential analysis of bacterial communities in the soil grown with *H. cordata* under different fertilization regimens. **(A)** Volcano plots indicating differential ASV changes between different treatments. **(B)** Histogram of linear discriminant analysis (LDA) Effect Size (LEfSe) showing taxa with log10 (LDA scores) ≥ 3.5.

The volcano plots at ASV level indicated that some taxa were enriched but others depleted after fertilization (Figure 2A). Compared to CK, only three ASVs were enriched while the other three were depleted after applying the Cd-immobilizing bacterial agent; 159 ASVs were enriched whereas 127 were depleted in FEMB; 118 ASVs were enriched but 119 were depleted in FE; 185 ASVs were enriched while 146 were depleted in FCMB; and only 36 ASVs were enriched whereas 54 ASVs were depleted in FC.

The LDA Effective Size (LEfSe) histogram (Figure 2B) demonstrated dramatic changes in these bacterial communities among different fertilized soils. The LDA scores of 34 bacterial taxa in all soil samples (including 4 in CK, 12 in FEMB, 7 in FE, 2 in FCMB, 9 in FC but none in MB) were higher than 3.5. These taxa biomarkers were fully diverse in different fertilized soils, e.g., *Bacteroidetes*, *Nitrosomonadaceae*, *Actinobacteria*, *Nitrospirae*, and *Pseudolabrys* were most prominent in FEMB, FE, FCMB, FC, and CK, respectively.

### Community Dissimilarity and Correlations With Environmental Factors

**FIGURE 3:**
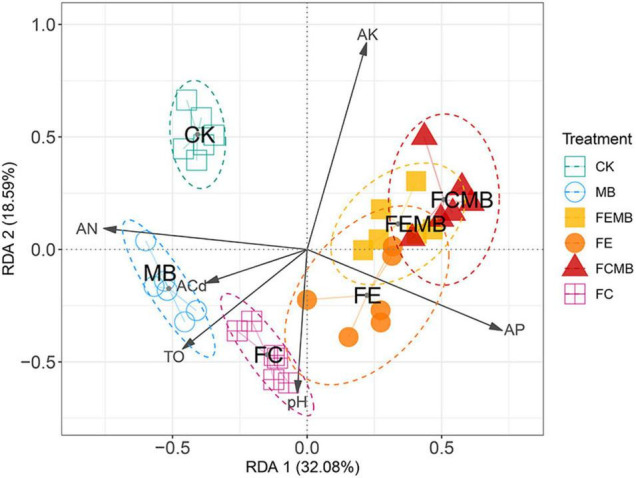
The Bray–Curtis distance-based redundancy analysis (bcRDA) of bacterial ASVs and characteristics of the soil grown with *H. cordata* under different fertilization regimens. Soil variables: pH, AN (available nitrogen), AP (available phosphorus), AK (available potassium), ACd (available Cd), and TO (total organic matter).

The redundancy analysis (RDA) revealed the roles of the environmental factors in driving the community changes (Figure 3). The RDA biplot, with its first and second axis explaining 32.08% and 18.59% of the total variance, showed that there was a significant difference in community composition among the six groups, as indicated by a good separation among them. All the five experimental groups showed distinctive differences from the control group. An obvious community dissimilarity was found between the group applied with Cd-immobilizing inoculant alone (MB) and those with a combination of the inoculant and other fermentative fertilizers (FCMB and FEMB). The combination of the bio-based fertilizers with the bacterial inoculant led to considerable community changes compared to the application of either the inoculant or the fermentative fertilizers. The length of the arrows indicated that the correlations between the environmental factors and the community structure were in the following descending order: available K > available P > available N > TO > pH > available Cd.

### Correlations Among Bacterial Communities in the Soil of *H. cordata*

**FIGURE 4:**
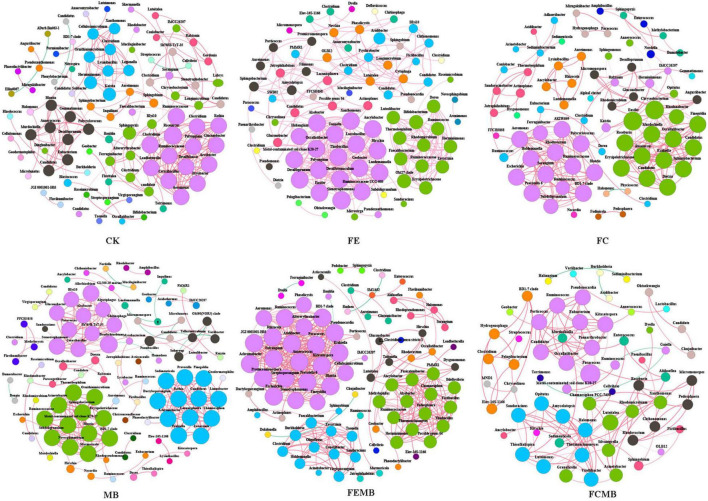
Co-occurrence network of bacterial communities at genus level in the soil grown with *H. cordata* under different fertilization regimens. Different colors of nodes in networks refer to different modularity classes. The size of each node reflects the degree of connection. Red lines represent positive correlations and green lines represent negative correlations.

The co-occurrence network (Figure 4) showed significant changes in the correlations of bacterial communities under different fertilization regimens. The microbial co-occurrence network of CK included 94 nodes and 219 edges with 98.63% positive correlations, while that of MB contained 102 nodes and 241 edges with 95.44% positive correlations. When the Cd-immobilizing bacterial agent was mixed with the fermentative edible fungi residue, the microbial co-occurrence network in soil had 90 nodes and 423 edges with 99.05% positive correlations, whereas only 85 nodes and 311 edges with 100% positive correlations were found when applied with fermentative edible fungi residue. However, when the Cd-immobilizing bacterial agent was applied in combination with the fermentative cow dung, the microbial metal co-occurrence network in soil contained 78 nodes and 240 edges with 99.58% positive correlations, while 64 nodes and 132 edges with 96.21 positive correlations were observed on the network when applied with fermentative cow dung. For the co-occurrence networks of fertilization treatment without the Cd-immobilizing bacterial agent, the nodes were clustered into 21 modules for CK, 15 modules for FE, and 14 modules for FC. When the Cd-immobilizing bacterial agent was applied, the nodes were clustered into 26, 16, and 15 modules for MB, FEMB, and FCMB, respectively. The average path lengths of the correlation networks of MB (1.32), FEMB (1.22), and FCMB (1.59) were lower than those of the fertilization treatments without the inoculant (CK, 1.84; FE, 1.33; and FC, 1.72). Except for the treatment using fermentative edible fungi residue as organic fertilizer, the network clustering coefficients of other two fertilization treatments with inoculant addition (MB, 0.97 and FCMB, 0.93) were higher than those without the inoculant (CK, 0.91 and FC, 0.90). However, applying the immobilizing inoculant alone (MB, 4.73) yielded higher network average degree than the non-fertilization group (CK, 4.66). The application of the fermentative edible fungi residue with the Cd-immobilizing inoculant (FEMB, 1.22) led to lower average degree than applying fermentative edible fungi residue alone (FE, 1.33). Similarly, the average degree of the application of fermentative cow dung with the inoculant (FCMB, 1.59) was lower than that with only fermentative edible fungi residue (FC, 1.72).

The betweenness centrality value indicated fully different keystone genera among the microbial communities in the field soil of *H. cordata*. According to this parameter, *Sphingobium*, *Herminiimonas*, *Sphingopyxis*, *Clostridium*, *Roseiarcus*, *Sphingobacterium*, *Sorangium*, *Aureimonas*, *Inquilinus*, and *Chryseobacterium* (ranging 50–16) were identified as the top ten keystone bacterial genera in CK. When only the Cd-immobilizing inoculant (MB) was applied into the *H. cordata field*, the top ten keystone genera were changed into *Bradyrhizobium*, *Allorhizobium*, *Mycobacterium*, *Stenotrophomonas*, *Hirschia*, *Paenibacillus*, *Nocardia*, *Luedemannella*, *Aureimonas*, and *Bosea* (ranging 27–1). In the field where only the fermentative edible fungi residue (FE) was applied, the top ten keystone genera consisted of OM27 clade, *Microvirga*, *Aridibacter*, *Geobacter*, *Bifidobacterium*, *Chryseolinea*, *Amycolatopsis*, *Lactobacillus*, *Sphingobacterium*, and *Stenotrophomonas* (ranging 36–1), whereas the top teen keystone genera in the field applied with fermentative edible fungi residue with the bacterial inoculant (FEMB) were changed into *Finegoldia*, *Stenotrophomonas*, *Burkholderia*, *Faecalibacterium*, *Sphingobium*, *Luteitalea*, *Altererythrobacter*, *Geobacter*, *Polyangium*, and *Occallatibacter* (ranging 23–1). When fermentative edible fungi residue was used in the *H. cordata* field (FC), the top ten keystone genera were *Polycyclovorans*, *Rhodanobacter*, *Nordella*, *Ensifer*, *Ruminococcus*, *Caulobacter*, *Sphingobacterium*, *Adhaeribacter*, *Candidatus*, and *Herminiimonas* (ranging 52–7.5), while they were replaced by only the following five genera of *Paracoccus*, *Luteitalea*, *Fictibacillus*, *Sphingobium*, and *Ruminococcus* (ranging 35–5) in the treatment using the combination of fermentative edible fungi residue and Cd-immobilizing inoculant.

### Prediction of Bacterial Ecological Functions in *H. cordata* Soil

**FIGURE 5:**
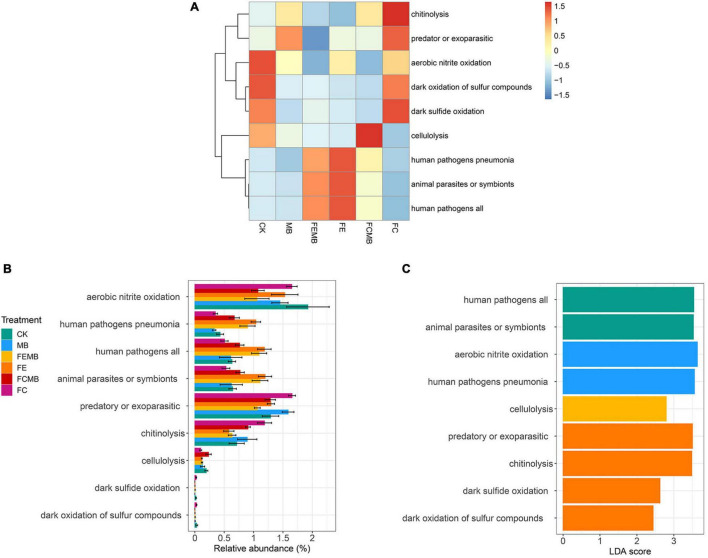
Microbial functional diversity (FAPROTAX) in the field grown with *H. cordata* under different fertilization regimens. **(A)** Relative abundance heatmap of the functions. **(B)** Relative abundance barplot of the functions. **(C)** Linear discriminant analysis (LDA) showing the prominent functions.

The FAPROTAX functional prediction revealed that there was a huge difference in microbial functions between CK and other five treatment groups (Figure 5A). Only several common functions including interspecies symbiosis (pathogens and parasites), soil nitrogen cycling (nitrite oxidation), biodegradation (chitinolytic and cellulolytic processes) and soil sulfur cycling (sulfur/sulfide oxidation) were significantly different (*p* < 0.05) in most soil microorganisms. The control soil was rich in aerobic nitrite oxidation, dark oxidation of sulfur compounds, dark sulfide oxidation, and cellulolysis. Fermentative edible fungi residue-treated soils (FE and FEMB) were rich in human pathogens and animal parasites. The soil with fermentative cow dung mixed with Cd-immobilizing bacterial inoculant (FCMB) contained a high proportion of cellulolysis function, while that with only fermentative cow dung (FC) showed a completely different pattern of the functional profiles, which featured chitinolysis, predatory or exoparasitic, aerobic nitrite oxidation, and sulfur/sulfide oxidation functions. The quantitative analysis visualized by the relative abundance plot showed that the top overall abundance of microbial functions for all the six groups was aerobic nitrite oxidation, followed by predatory or exoparasitic function (Figure 5B). Compared to CK, all the other five treatment groups had a lower amount of aerobic nitrite oxidation function. The amounts of sulfur and sulfide-related functions were negligible compared to the other seven functions. Compared to the application of fermentative edible fungi residue alone, the combination of the Cd-immobilizing bacterial inoculant and fermentative edible fungi residue reduced functions including aerobic nitrite oxidation, human pathogens, animal parasites, and predatory or exoparasitic, while slightly increasing chitinolytic function. Compared to the application of fermentative cow dung alone, the inclusion of the Cd-immobilizing inoculant induced a reduction in aerobic nitrite oxidation, predatory or exoparasitic, and chitinolytic functions, but an increase in human pathogens, animal parasites, and cellulolysis. The linear discriminant analysis (LDA) (Figure 5C) showed that human pathogen and animal parasite-related functions attained significance in FEMB; aerobic nitrite oxidation and human pathogens were significant in FE; cellulolysis was prominent in FCMB; predatory or parasitic, chitinolytic, sulfur, and sulfide oxidation functions were the significant biomarkers in FC.

## Discussion

In our study, it was shown that the Cd-immobilizing bacterial inoculant can effectively prevent the accumulation of Cd in the roots of *H. cordata*, even when this agent was applied alone. This indicated that the microbial agent survived in the Cd-polluted soil and affected the uptake of Cd for the plant. There was very limited information as to the bioaccumulation of Cd and other heavy metals by *H. cordata*. Interestingly, all relevant studies showed that this plant was able to accumulate heavy metals (with or without microbial additives) and could play a role in the phytoremediation of metal-polluted lands. One study showed that *H. cordata* accumulated more than 1,000 ppm Pb in the roots and the inoculation of a *Bacillus* strain facilitated the transportation of Pb from underground to aboveground parts (Liu et al., 2018). Another pot experiment study revealed that the inoculation of a *Serratia marcescens* strain was able to enhance the uptake of Cd by *H. cordata* in both aboveground (by 34.48%) and underground parts (by 59.13%) (Chen et al., 2019). However, our study was performed from a completely different perspective, which featured the prevention of Cd uptake in plants by dint of the immobilizing ability of the bacterial agent. The abovementioned study also found that the growth of this plant was not drastically influenced by the presence of high levels of Cd (5, 50, and 100 mg kg^–1^ Cd in soil), which are way much higher than those in our work (Chen et al., 2019). However, the application of bio-organic fertilizers can promote the growth of *H. cordata* plants in this study. It is known that *in situ* metal immobilization occurs when microbes release inorganic and organic compounds into the soil, causing sorption of metals and metalloids by the reactions of various microbe-produced functional groups such as amine, carboxyl, hydroxyl, and sulfhydryl contained in organic molecules (e.g., chitin, humic, and lignin substances) (Zhou and Haynes, 2010). Other organic composts such as biosolids and animal (livestock and poultry) manure products have been traditionally applied in agriculture and they showed pronounced abilities to reduce the bioavailability of metal(loid)s to plants (Bolan et al., 2014). However, the present study did not corroborate this, as shown by the significant increase of Cd in the roots of the plants. On the contrary, it can be possibly inferred from our results that since more Cd was absorbed into the roots, the bioavailability of Cd was actually increased after the application of fermentative edible fungi residue (FE) and fermentative cow dung (FC). Studies showed that low bioavailability fractions of heavy metals including Cd, Cu, Ni, Pb, and Zn were increased as a result of composting (Smith, 2009; Shehata et al., 2019), and the mechanisms were closely related to organic acids released during certain stages of the process (Ingelmo et al., 2012; Irshad et al., 2014). Likewise, edible fungi residue was also reported to be able to induce high amounts of humus and organic compounds in soil environment (Li et al., 2020), which could cause the same effects as cow dung on the bioavailability of metals. It should be noted that, in our study, the combined application of the abovementioned bio-based amendments with the Cd-immobilizing agent reduced >20% of the uptake of Cd by the plant. This provided solid evidence to the fact that our beneficial microbial inoculant was able to perform outstanding Cd immobilization in the Cd-contaminated farm soil. In our study, all the four Cd-immobilizing strains were identified as belonging to the *Bacillus* genus. It is widely reported that many *Bacillus* species were very efficient in the immobilization of Cd through the mechanism of MICP and have been used as microbial soil amendments for the remediation of Cd-contaminated soil (Wang et al., 2014; Fang et al., 2021). Although the application of the microbial agent alone and the combination with the bio-based amendments showed no obvious signs of increase of total nitrogen uptake by the plant, they significantly (*p* > 0.05) elevated total and available nitrogen in the Cd-polluted soil. Because nitrogen is an important indicator of soil fertility (Zhong et al., 2010), the increase of N in the Cd-polluted soil implied that the Cd-immobilizing microbial agent was more competent in the improvement of soil quality when combined with organic fertilizers. Our results were in accordance with others where the combined application of different organic fertilizers and microbial agents greatly improved the agronomic traits of crops (e.g., *Elymus dahuricus, Nicotiana tabacum*, and strawberry plants) and soil conditions (Esitken et al., 2010; Liu Y. et al., 2013; Li et al., 2021; Tariq et al., 2021).

The high-throughput Illumina sequencing results indicated that the microbial α-diversity remained almost unchanged when the microbial inoculant was applied alone. The application of fermentative edible fungi residue (FE) apparently reduced while that of fermentative cow dung (FC) increased the microbial α-diversity compared to the non-fertilized control (CK). This may be due to the fact that animal manure itself contains microorganisms and more complicated organic matters compared to spent mushroom substrates (Aira et al., 2015; Xie et al., 2021). Interestingly, the introduction of the Cd-immobilizing bacterial agent to the fermentative cow dung (FCMB) reduced 16.7% of the community richness index (Chao1 estimator), while a slight reduction was found when the inoculant was combined with fermentative edible fungi residue (FEMB). Chao1 index estimates the abundance of microbial communities within the sample and the reduction of it means that the microbial populations in FCMB was reduced in number (Chodak et al., 2013). This phenomenon was probably due to the antagonistic effects of the newly introduced microbial strains on the native microbiome contained in the animal manure. This assumption has been confirmed by numerous studies that microbial soil amendments and bio-organic fertilizers can successfully prevent or reduce bacterial wilt disease in tobacco plants and can have a huge influence on the plant-associated soil microbial populations (Wu et al., 2014; Shen et al., 2015; Wu B. et al., 2016; Li X. et al., 2017; Ma et al., 2018). The combined application of the bacterial agent with both fungi residue and cow dung increased the abundance of *Actinobacteria and Bacteroidetes*. The results that more ASVs were significantly enriched and depleted in FCMB and FEMB than in the control group implied that there were increased microbial turnovers following the combined application of bio-based amendments and the bacterial agent. This may indicate that although the microbial abundance was reduced, the communities were obviously changed. The bcRDA plot clearly showed that there was a marked difference between the treatments with the combined applications (FCMB and FEMB) and those with single fertilization regimen (CK, MB, FC, and FE). This apparently indicated that β-diversity of the plant-associated microbial communities was greatly enhanced after the combined fertilization method was utilized. It also showed that the combination of the bacterial agent with both organic fertilizers (FCMB and FEMB) led to similar microbial composition, whereas the microbial structure between FC and FE showed a marked difference. This may point to the fact that the bacterial agent was competent in the alteration of microbial populations when applied together with organic fertilizers. The increase of diversity and reduction of abundance may be the result of an increase of certain taxa biomarkers (i.e., *Bacteroidetes* in FEMB; *Actinobacteria* in FCMB) discovered using the LDA analysis. The environmental-community structure analysis further identified that available K, P, and N, which are basic soil nutrient parameters, had a greater influence on the community structure dissimilarity compared to total organic matter and available Cd in the soil. Based on the analysis of the angles between environmental factors and sample points, it can be inferred that available nitrogen (AN) was positively correlated with the microbial population in MB but was negatively correlated with that in the co-application groups (FCMB and FEMB). Interestingly, available K and P showed negative correlations with MB but positive correlations with FCMB and FEMB. Although available NPK had a much bigger influence on the overall structure of the microbial population, we cannot directly conclude from the bcRDA plot as to whether they can be increased or reduced as a result of the application of the bacterial agent and organic fertilizers, since they might be the cause of the changes of microbial population.

The co-occurrence network analysis is a very effective tool for the reflection of complexities of the microbial associations under different fertilization regimens (Vendruscolo et al., 2020; Yuan et al., 2021). More edges (indicating co-occurring relationships) and less nodes (indicating taxa abundance) were found in the treatments with combined fertilization compared to the treatments with the bio-based amendments alone, which again implied that the introduction of the Cd-immobilizing inoculant reduced the abundance while at the same time increasing the complex or diversity of the *in situ* microbial communities. The smaller average path lengths and higher clustering coefficients in the networks of FCMB and FEMB suggested that the bacterial agent enhanced ecological relationships among the microbial populations. The lower average degree values in FCMB and FEMB also implied that the addition of the microbial inoculant led to reduced connections among the individuals as a result of a decrease in abundance. Keystone taxa, which reflect the important roles of certain microbial species play in an ecological network, can be identified using the betweenness centrality value as a measure (Banerjee et al., 2018, 2019; Zeng et al., 2019). Our result showed that the top 10 taxa in the co-occurrence networks varied significantly under different fertilization regimens. This could possibly imply that the microbial agent exercised a prominent influence on the ecological relationships among the communities in the soil.

The FAPROTAX method has been widely used in the prediction of soil microbial functions related to biogeochemical processes (Zhou et al., 2020). Our study revealed that there was a significant increase in the functions pertaining to human pathogen and animal symbiont functions after fermentative edible fungi residue was applied alone (FE), and the cause of this is unknown. However, the addition of the microbial agent slightly reduced the presence of those functions, which could potentially suggest that the microbial agent was able to suppress some harmful microorganisms contained in the fermentative edible fungi residue. On the contrary, the combination of the bacterial agent with the fermentative cow dung could be able to increase those functions, which may be the result of synergism between the microbial agent and the native microorganisms contained in the cow dung. As for other functions, the application of the Cd-immobilizing bacterial agent invariably reduced the aerobic nitrite oxidation function, and this can clearly indicate that there was a reduction of the abundance of nitrite oxidizing bacteria (NOB), which catalyze the second step of nitrification, converting nitrite to nitrate (Daims et al., 2016; Baskaran et al., 2020). NOB species are mainly found in the genera of *Nitrobacter*, *Nitrococcus*, *Nitrospira*, and *Nitrospina*, which play an important role in the process of nitrification after ammonium is firstly oxidized to nitrite by ammonia-oxidizing bacteria (AOB) (Spieck and Lipski, 2011; Yao and Peng, 2017).

## Conclusion

The present study took a close examination of the effects on the plant *H. cordata* and its microbial communities under different fertilization regimens in a Cd-polluted field. Our research found that both the application of the Cd-immobilizing bacterial agent alone and the combined application of bio-based soil amendments and the bacterial agent can effectively reduce the uptake of Cd by the plant. The combined fertilization can raise soil nitrogen level. After the combined fertilization regimen was introduced, there was a significant shift of microbial communities: the microbial populations tended to be homogeneous with reduced microbial abundance and increased diversity. The combined application of the bacterial agent with fermentative edible fungi residue and cow dung significantly increased the abundances of *Actinobacteria* and *Bacteroidetes*, respectively. The introduction of the microbial agent could potentially suppress human pathogenic microorganisms in the field fertilized with edible fungi residue. The microbial agent can also reduce the nitrite oxidation function in the soil when applied alone or with the bio-based soil amendments. Our study thus highlights the beneficial effects of the Cd-immobilizing bacterial inoculant on *H. cordata* and provides a better understanding of the microbial changes induced by combined fertilization using the microbial agent and natural soil amendments.

## Data Availability Statement

The original contributions presented in the study are included in the article/Supplementary Material, further inquiries can be directed to the corresponding author.

## Author Contributions

XY, MY, and YC designed the study and wrote the manuscript. XY, MY, YC, HL, JZ, JL, QX, and LZe performed the experiments. XY, MY, YG, KZ, and LZo analyzed the data. QC, MM, and LZo reviewed and edited the manuscript. XY and YC funded and supervised the experiments. All authors participated in the interpretation and discussion of results and contributed to the article.

## Conflict of Interest

The authors declare that the research was conducted in the absence of any commercial or financial relationships that could be construed as a potential conflict of interest.

## Publisher’s Note

All claims expressed in this article are solely those of the authors and do not necessarily represent those of their affiliated organizations, or those of the publisher, the editors and the reviewers. Any product that may be evaluated in this article, or claim that may be made by its manufacturer, is not guaranteed or endorsed by the publisher.

## Footnotes

1
http://egcloud.cib.cn/

2
https://gephi.org/

## Supplementary Material

Supplementary Figure 1 Characteristics of bacterial communities in the soil before and after biofertilizer treatments. **(A)** Volcano plots indicating differential ASV changes before and after different treatments. **(B)** Relative abundance heat map of the top 12 phyla for all treatments. **(C)** Co-occurrence network of bacterial communities at genus level in the soil before biofertilizer treatments.

Supplementary Table 1 Amplicon sequence variants (ASVs) of 16S rRNA gene for the cadmium-contaminated *Houttuynia cordata* field soil in different biofertilizer treatments.

Supplementary Table 2 Alpha-diversity of bacterial communities in *Houttuynia cordata* farm soil before the application of bacteria and organic fertilizers.
