# Supplementary material for: Effects of Co-application of Cadmium-Immobilizing Bacteria and Organic Fertilizers on Houttuynia cordata and Microbial Communities in a Cadmium-Contaminated Field
Source: Front Microbiol. 2022 May 6;12:809834. doi: 10.3389/fmicb.2021.809834 (PMC9122265; doi:10.3389/fmicb.2021.809834)
Supplement: Supplementary Table 2 — Alpha-diversity of bacterial communities in Houttuynia cordata farm soil before the application of bacteria and organic fertilizers. [file Table_2.docx]

Table 2 Alpha-diversity of bacterial communities in *Houttuynia cordata* farm soil before the application of bacteria and organic fertilizers

| Diversity index | Observed ASVs | Good's coverage | Chao1 | ACE | Shannon | Simpson |
| --- | --- | --- | --- | --- | --- | --- |
| Value | 1869 ± 222 | 0.92 ± 0.01 | 2282 ± 255 | 2457 ± 307 | 9.59 ± 0.32 | 0.996 ± 0.002 |
